# Review and publication of protocol submissions to *Trials* – what have we learned in 10 years?

**DOI:** 10.1186/s13063-016-1743-0

**Published:** 2016-12-16

**Authors:** Tianjing Li, Isabelle Boutron, Rustam Al-Shahi Salman, Erik Cobo, Ella Flemyng, Jeremy M. Grimshaw, Douglas G. Altman

**Affiliations:** 1 Department of Epidemiology, Johns Hopkins Bloomberg School of Public Health, 615 North Wolfe Street, Room E6011, Baltimore, MD 21205 USA; 2 University Paris Descartes, Inserm Methods Team, CRESS-UMR 1153, Paris, France; 3 Centre d’épidémiologie Clinique, Hôpital Hôtel-Dieu, 1 place du Parvis Notre-Dame, Paris, 75004 France; 4 Centre for Clinical Brain Sciences, University of Edinburgh, Chancellor’s Building, 49 Little France Crescent, Edinburgh, EH16 4SB UK; 5 Statistics and Operations Research Department, Barcelona Tech, Carrer de Jordi Girona 1, C5-213, 08034 Barcelona, Spain; 6 BioMed Central, 236 Gray’s Inn Road, London, WC1X 8HB UK; 7 Ottawa Hospital Research Institute, The Ottawa Hospital - General Campus, 501 Smyth Road, Box 711, Ottawa, ON K1H 8L6 Canada; 8 Centre for Statistics in Medicine, University of Oxford, Botnar Research Centre, Windmill Road, Oxford, OX3 7LD UK

## Abstract

*Trials* has 10 years of experience in providing open access publication of protocols for randomised controlled trials. In this editorial, the senior editors and editors-in-chief of *Trials* discuss editorial issues regarding managing trial protocol submissions, including the content and format of the protocol, timing of submission, approaches to tracking protocol amendments, and the purpose of peer reviewing a protocol submission. With the clarification and guidance provided, we hope we can make the process of publishing trial protocols more efficient and useful to trial investigators and readers.

The importance of establishing a protocol at the outset of every clinical trial is unarguable [1–9]; however, the availability and completeness of protocols and their consistency with the final study report have been poor [10–17]. Making protocols publicly available will contribute to improving the transparency of research and will enable researchers and prospective participants to learn about trials that are underway. Protocol availability also enables systematic reviewers, sponsors, regulators and others to understand the scientific rigor of the design and results, as well as to compare what was intended with what was described in the reports of trials in order to assess possible reporting bias [7–20].

Journals and trial registries are part of the solution to these problems because they provide a platform for making trial protocols publicly available [8]. Beginning in 2006, *Trials* pioneered open access publication of protocols for randomised controlled trials. Other journals have taken similar approaches [21–23], but more than half of published trial protocols have been published in *Trials* [24]. From 2006 to November 2016, *Trials* published over 2100 protocols. In general, though at the editor’s discretion, those protocols with previous full external peer review as part of the process of receiving a grant from a major external funding body or ethics approval were published with peer review from a member of the editorial board [25], with the remainder undergoing full peer review. *Trials* provides authors with guidance [25], but uncertainties remain.

The roles of journals, journal editors and peer reviewers are not well defined when a trial protocol, as a stand-alone document, is submitted to a journal for consideration for publication. How much detail should be included in the protocol? What is the ‘right’ moment that the protocol should be made publicly available? How should subsequent protocol amendments be documented and linked to the published version of the protocol? What are appropriate peer review and editorial comments and suggestions when the trial is already underway, especially if the protocol has already been approved by the sponsor and the institutional review board (IRB) or research ethics committee (REC)? Herein we, the senior editors and editors-in-chief of *Trials*, discuss editorial issues regarding managing protocols submitted for publication and provide guidance for future protocol submissions to *Trials*.

## What is expected in a protocol submission?

Articles that describe protocols are different from traditional trial ‘design articles’ and from the Methods sections of articles that report trial results. Design articles typically follow the format of a research report, including Background, Methods, Results and Discussion sections. We are aware of two types of design articles. Design articles published in clinical journals typically include an abbreviated description of the protocol, are published after enrolment has been completed, and report baseline characteristics of participants in the Results section. Design articles published in methodological journals focus on new, uncommon or challenging aspects of the trial design or procedures; protocol details are included only as necessary to understand the issues [26]. Here readers can find examples of two design articles, one for each type, arising from the same trial [27, 28].

In contrast, a protocol, as defined in the Standard Protocol Items: Recommendations for Interventional Trials (SPIRIT) statement, is

*a document that provides sufficient detail to enable understanding of the background, rationale, objectives, study population, interventions, methods, statistical analyses, ethical considerations, dissemination plans, and administration of the trial; replication of key aspects of trial methods and conduct; and appraisal of the trial’s scientific and ethical rigour from ethics approval to dissemination of results.* ([29]; p. 203)

Typically, each element above is described in a distinct section, and no performance or scientific results are included.

So, a protocol should provide sufficient details for readers to understand the general design and major operating features of the trial. However, ‘this document is generally distinguished from the study manual of operations by the absence of detailed instructions needed for the actual application of treatment and data collection in the trial’ [30]. Following the SPIRIT guideline is one way to strike a balance between comprehensiveness and conciseness [29, 31]. *Trials* endorses SPIRIT and requires authors to submit a completed SPIRIT checklist as an Appendix file and also to include a SPIRIT figure that depicts the schedule of enrolment, interventions and assessments within the main body of the article. The *Trials* Editorial Office is asked to verify the fulfilment of this requirement before assigning a new submission to an editor.

When reviewing protocol submissions, we have noticed that the completeness and understanding of the reporting of items in the SPIRIT checklist could be better among authors. In some submissions, the authors have entered on the SPIRIT checklist ‘not applicable’ for many critical items that in our view are applicable to any randomised controlled trial (e.g., Item 6c. Explanation for choice of comparators [29]). One possibility is that what the item refers to was not done in the trial; another is that the authors see the description as unnecessary. When an item is truly ‘not applicable’, it is to the authors’ advantage to provide a succinct explanation so that the readers understand the rationale for not addressing the item on the SPIRIT checklist in their protocol. For items 16b and 16c concerning allocation concealment mechanism and implementation [29], some authors have mistakenly referred to the section of the protocol that describes blinding. We encourage authors to read the SPIRIT explanation and elaboration document [31], which will help them improve their trial design, save time in publishing the manuscript and enhance transparency. In addition, the items on the SPIRIT checklist can be used as a general guide to organising the contents of the protocol.

## When to submit a protocol for publication

Procedures in trials almost always change over the course of the trial for scientific and logistic reasons. Some of these changes are trivial, such as modification of an item on a data collection form to improve clarity, but others might be important enough to trigger a protocol amendment. For example, the IRB/REC, the data monitoring committee or the sponsor may recommend collecting safety outcomes that were unknown to the investigators at the outset of the trial, adding a new clinical site to achieve the target recruitment goal, or adopting a new statistical method to analyse the data. In general, protocol amendments should be limited to the most relevant and critical ones that affect the trial design and data collection procedure. The final version of the protocol, which tracks all protocol amendments, provides a fuller and more accurate account of the trial design and implementation than the version submitted to the sponsor for funding, to the IRB/REC for ethics approval, or to the data monitoring committee before enrolment begins.

We encourage investigators to submit their trial protocol for publication at an appropriate stage during the conduct of the trial; almost always this should be before recruitment is complete. In a ‘Trial status’ section, the authors should report the protocol version number and date, the date on which recruitment began, and the approximate date when recruitment will be completed. Authors should also describe any protocol amendments that were made after trial commencement, along with the rationale for such changes.

When amendments are made to the protocol after its publication in *Trials*, we encourage the authors to submit an ‘Update’, an article type that enables authors to modify the published record. Depending on the particular amendments, the Update article is generally free to submit and is linked back to the original article. Authors should bear in mind that the credibility of a trial can be damaged when changes are not reported, which also complicates the assessment of a trial report at a later date if inconsistencies between the protocol and the report are identified.

As of November 2016, *Trials* had published 38 updates, two-thirds of which are statistical analysis plans (SAPs). We do not consider SAPs as protocol amendments. A SAP describes the planned analysis for a clinical trial. It may be written as a separate document after finishing the protocol ([30]; p. 257). SAPs are required for trials on regulated products, a subset of all trials. The International Conference on Harmonisation defines a SAP as ‘a document that contains a more technical and detailed elaboration of the principal features of the analysis described in the protocol, and includes detailed procedures for executing the statistical analysis of the primary and secondary variables and other data’ ([32]; p. 35). At most pharmaceutical companies, the trial statisticians write the SAP following a standardised template; general guidance is under development [33]. SAPs typically include sections providing a brief description of the study design, a description of the analysis population, data-handling rules, precise analyses to be done and statistical methods to be used for each, as well as shells for planned tables, figures and listings [32]. We encourage trial investigators to submit SAPs, regardless of funding source, and link them to the trial protocols when they become available.

## What is the purpose of peer review of trial protocol submissions to *Trials*?

One essential purpose of peer review of a protocol submission is to improve clarity of reporting. Because the protocol is more than a list of items (e.g., as in ClinicalTrials.gov or the World Health Organisation International Clinical Trials Registry Platform), it is reasonable for peer reviewers and editors to ask the authors to provide a fuller and richer narrative of certain aspects of the design or, in some cases, to shorten overly long descriptions, to clarify unclear and seemingly inconsistent descriptions, and to provide justifications and rationales for aspects of the chosen design.

The comments made by peer reviewers and editors should be geared towards improving the reporting rather than commenting on the design. It is possible, however, that the peer reviewers and editors may raise concerns over the design. These suggestions, although potentially valuable, are not typically within the purview of a journal and usually cannot be implemented by the investigators when the trial is already underway. When appropriate, we may ask the authors to discuss the issues and limitations raised by reviewers’ concerns. In addition, the readers of the final results paper can consider these potential limitations because the comments made by reviewers for *Trials* are open to the public and published alongside the article within the Open Peer Review reports. Finally, the editors may invite a commentary on the design of a trial or a set of trials.

Although our goal is to facilitate public availability of trial protocols so that readers can judge the trials’ scientific rigor, we reject some protocol submissions because of a lack of clarify in reporting, poor adherence to SPIRIT guidelines without justification, or serious concerns over the design that preclude replication of the trial by other researchers.

## Can we improve the process?

The turnaround time for providing the authors with the first decision on a protocol submission could be shortened if (1) the authors optimised the quality of reporting and submitted a manuscript that followed the journal’s instructions and requirements, (2) the editors and peer reviewers were familiar with the journal’s position on the issues stated above, (3) more of the trials community contributed to the journal as editors and peer reviewers, and (4) peer reviewers continued by providing constructive comments intended to improve the quality of reporting. Ultimately, *Trials* aims to increase value and reduce waste in the reporting of clinical trials [7]. The principal recommendations by Chan et al. for reducing inaccessible research (see box below) illustrate the value that investigators can derive from fully accessible and clear reporting of clinical trial protocols [7], as well as the role that *Trials* plays in facilitating this.

**Box:** Recommendations from Chan et al. for reducing inaccessible research in *The Lancet*’s Increasing Value, Reducing Waste series [7]

1. Institutions and funders should adopt performance metrics that recognise full dissemination of research and reuse of original datasets by external researchers
2. Investigators, funders, sponsors, regulators, research ethics committees, and journals should systematically develop and adopt standards for the content of study protocols and full study reports, and for data sharing practices
3. Funders, sponsors, regulators, research ethics committees, journals, and legislators should endorse and enforce study registration policies, wide availability of full study information, and sharing of participant-level data for all health research

We encourage researchers to continue sending the protocols for their randomised controlled trials to *Trials* for consideration for publication. We also encourage researchers who are committed to full and transparent reporting to join *Trials* as editors or peer reviewers. We will strive to make the process for publishing trial protocols more efficient and useful to trial investigators and readers.
